# Primary adenoid cystic carcinoma of the trachea: a report of two cases and literature review

**DOI:** 10.11604/pamj.2014.19.32.4878

**Published:** 2014-09-15

**Authors:** Mohammed El Marjany, Adil Arsalane, Hassan Sifat, Khalid Andaloussi, Mohamed Oukabli, Khalid Hadadi, El Hassan Kabiri, Hamid Mansouri

**Affiliations:** 1 Department of Radiation Oncology, Mohamed V Military Teaching Hospital, University Mohammed V Souissi, Rabat, Morocco; 2 Department of Thoracic Surgery, Mohamed V Military Teaching Hospital, University Mohamed V Souissi, Rabat, Morocco; 3 Department of Histopathology, Mohamed V Military Teaching Hospital, University Mohamed V Souissi, Rabat, Morocco

**Keywords:** Adenoid cystic carcinoma, trachea, tracheal resection, radiotherapy

## Abstract

Adenoid cystic carcinoma (ACC) of the trachea is rare, it represents 1% of all respiratory tract cancers. It's generally considered as a slow-growing, with pronlonged clinical course. Most patients present with dyspnea, and the symptoms often mimic those of asthma or chronic bronchitis Surgical resection is the mainstay of treatment often combined to radiotherapy because of close surgical margins. When surgery isn't possible, most tumors respond to radiotherapy alone wich often results in long periods of remission We report two cases of primary ACC of trachea: a 49 year old male presented a distal unresectable tracheal ACC treated with chemo-radiotherapy who developed a recurrence and died 7 years after the diagnosis. And a 50 years old female with a proximal tracheal tumor treated by surgical resection and end- to- end anastomosis followed by adjuvant radiotherapy. At 10 months follow-up, our patient shows no evidence of disease with negative histological findings.

## Introduction

Tumors of tracheal origin are exceedingly rare; the incidence of primary tracheal tumors is less than 0,2 per 100,000 persons per year [1]. Malignant tumors are more common than benign tumors and account for 60 to 83% in adults [2]. Squamous cell carcinoma and Adenoid cystic carcinoma (ACC) are the most frequent histologic types, accounting for approximately two-thirds of primary neoplasms of the air-way [3]. The clinical and pathologic features of ACC of the trachea; wich was formely named “cylindroma” and “adenoicystic carcinoma”, were initially reported in 1859 by Billroth [4]. It is usually found in younger patients and it appears to be unrelated to smoking, with an equal distribution in males and females [5]. Most ACC patients present with dyspnea, and the symptoms often mimic those of asthma or chronic bronchitis. The speed of growth is slow and the clinical course is relatively long, so it is generally considered to be a low grade-malignancy, but it tends to metastasize to distant sites and often recurs after a long interval. The primary management of tracheal ACC is surgical resection. However, resectability often becomes difficult if invasion of adjacent critical tissues; especially in patients with distal tracheal involvement [6, 7], or tumors are too large to permit surgery. In this situation radiotherapy is used in the postoperative setting or as the primary modality in unresectable disease. We herein report two cases of primary ACC of the trachea: the first patient presented a distal unresectable tracheal ACC treated with chemo radiotherapy who developed a reccurence, and the second patient with a proximal tracheal tumor treated by surgical resection followed by adjuvant radiotherapy.

## Patient and observation

### Case 1

**Figure 1:**
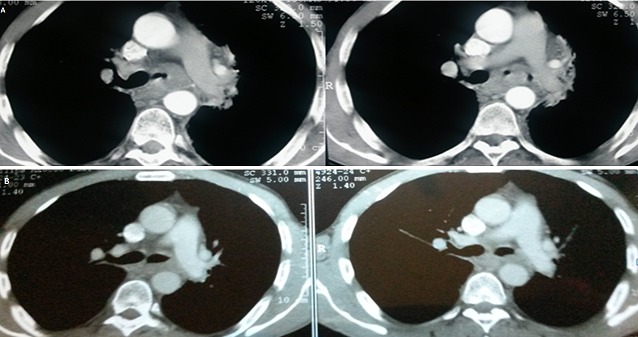
Transaxial CT scan showing a large polypoid intra-luminal mass involving the distal trachea and the carina, causing near total obliteration of the lumen. (A): Before radiotherapy, (B): After radiotherapy

A 49 year old male developed since 2 months dyspnea with dry cough and thoracic pain. He had smoked 1 pack/day cigarettes for 18 years and has been ex-smoker for 9 years. In November 2004, he was admitted with almost complete atelectasis of the left lung at the chest X-ray. Chest computed tomography (CT) was performed that showed soft tissue mass surrounding the trachea extending from the left pulmonary hilum through the left main bronchus to the carina. This tumor was in close contact with the descending aorta. Contrast-enhanced CT scans demonstrated no paratracheal adenopathy or pulmonary parenchymal disease (Figure 1). The patient underwent a bronchoscopy that showed mural irregularities extending from 15 mm above the carina into the left mainstem bronchus. The circumferential submucosal masses protruded and obstructed the lumen of the proximal left main-stem bronchus. Bronchoscopic biopsies of the mural lesions showed ACC infiltrating respiratory mucosa. Extended tracheal resection was considered, and neoadjuvant chemotherapy was delivered in order to achieve tumor reduction and allow eventual surgical excision. Scanner control performed after 4 cycles of vinorelbine -CDDP showed an unchanged aspect of the tumor.

A multidisciplinary team of medical, surgical and radiation oncologists made a decision to treat the patient with definitive radiotherapy. The patient received 66 Gray (Gy) of radiotherapy to the primary tumor volume in 33 fractions; 5 fractions per week with exclusion of the spinal cord at 46 Gy. Bronchoscopies performed 6 and 12 months after therapy showed a disappearance of the tumor mass of the left bronchus wich became permeable. The patient was lost sight of for 4 years. In july 2010, he was readmitted with a history of severe respiratory insufficiency at rest. Bronchoscopy revealed an obstructive and hemorrhagic tumor of the left mainstem bronchus invaded the carina, and the right main bronchus, wich prevented us to biopsy. Chest CT showed an extensive tumour regrowth in the mediastinum, with atelectasis of the left upper lobe, and mediastinal retraction to the left side. A palliative chemotherapy (based on carboplatine- paclitaxel) was programmed, but the patient died from tumor progression. The patient has survived 7 years from the first medical assessment.

### Case 2

**Figure 2:**
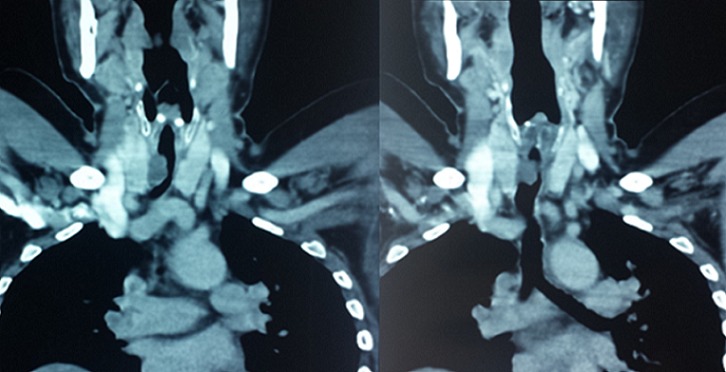
Coronal reformatted CT image showing a large polypoid intra-luminal mass arising in the trachea within 1 cm of the larynx, causing obliteration of 70% of the lumen

**Figure 3:**
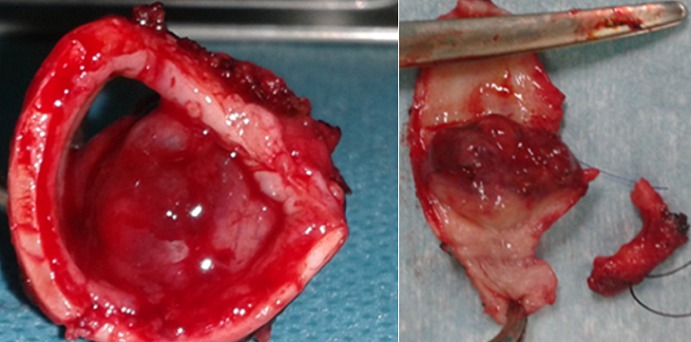
Resected specimen showing a polypoid growth pattern. The tumor was present at 1 cm below the larynx

**Figure 4:**
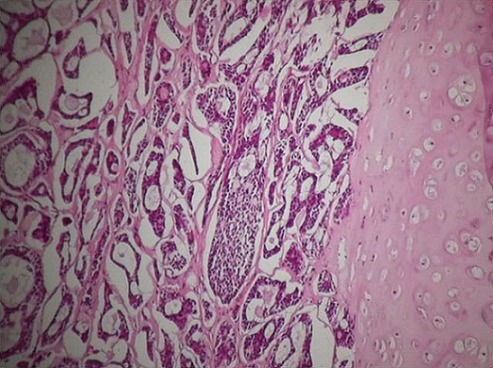
Histologic specimen showing adenoid cystic carcinoma mixed with tubular and cribriform pattern (H&E, original magnification x 10)

**Figure 5:**
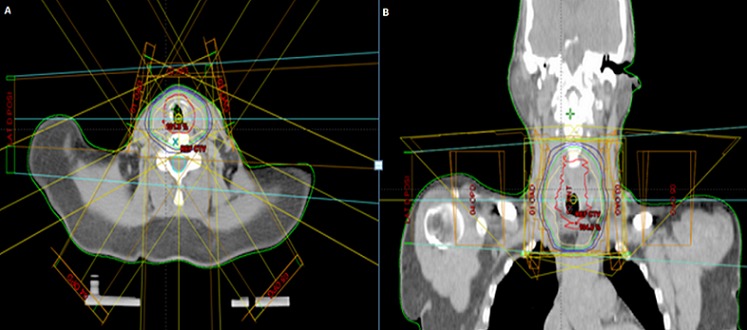
An axial (A) and coronal (B) figures shows the dose distribution of the radiation treatment plan. The plan uses five fields (anterior, right anterior oblique, left anterior oblique, right posterior oblique and left posterior oblique). Coloring wires shows the high dose (shown in yellow) and lower doses (shown in green and blue, respectively)

A 50 - year-old female, with no particular history; had complaints of nocturnal dyspnea, intermittent cough for three years and had been treated with various bronchodilators and anti-asthmatic medications without significant results; worsening dyspnea continued without improvement. Her investigations revealed a normal chest X-ray. Chest CT was performed that showed a large polypoid intra-luminal mass arising in the trachea within 1 cm of the larynx, causing obliteration of 70% of the lumen (Figure 2). A flexible-bronchoscopy demonstrated a polypoid tumor with a smooth surface; and demonstrated striking neovascularity. The tumor was localized on the anterior tracheal wall 1 cm below the larynx and lying on three cartilaginous rings. Histopathological diagnosis was ACC of the trachea. A resection of the first four tracheal rings and end-to-end anastomosis of the trachea was carried out (Figure 3). Histologically, ACC was diagnosed (Figure 4). The surgical margins were not invaded but the upper margin was at 3 mm. Postoperative locoregional conformal radiation around the anastmosis, with a total dose of 50 Gy, was delivred in 25 fractions, 4 weeks after surgery. Figure 5 shows the radiation treatment plan. Ten months after the operation, the patient has remained well.

## Discussion

Previously called cylindroma or adenocystic carcinoma, ACC of the trachea is a relatively rare occurrence. The incidence of tracheal tumors is less than 0,2 per 100,000 people per year [1]; only 10% of these cases are ACC [8]. ACCs have been reported without sex predilection in patients in the fifth decade of life; our patients are 49 and 50 years old; and smoking does not affect the incidence of ACC [9]. Patients with ACC usually present with symptoms such as coughing, wheezing and dyspnea and are often treated for asthma for months to years before being correctly diagnosed [1], as our second patient. ACC is a nonencapsulated tumor, it spreads most commonly by direct extension, submucosal or perineural invasion, in transverse and longitudinal planes [7]. Lymphatic spread is uncommon. More than 50% of patients with tracheal ACC have hematogenous metastases. Pulmonary metastases are the most commun and can remain asymptomatic for many years [9]. Metastases to the brain, bone, liver, kidney, skin, abdomen; and heart have also been reported [1, 2, 5]. On CT, the tumor infiltration in the submucosa manifests as an intraluminal mass of soft-tissue attenuation with extension through the tracheal wall, and a diffuse or circumferential wall thickening of the trachea, a soft-tissue mass filling the airway, or a homogeneous mass encircling the trachea with wall thickening in the transverse and longitudinal planes [2]. MRI because of its multiplanarity and better tissue characterization can better define the extent of the submucosal infiltration, and local mediastinal invasion than CT that may influence resectability [9].

Treatment options include surgery alone, radiation therapy alone; or a combination. The ideal treatment of ACC is primary resection and end-to-end anastomosis when possible. A surgical resection has been thought to be the most favourable procedure to control localized lesions. The median survival time of resected patients was reported to range from 7,5years to 118 months [10]. Because they tend to infiltrate along the airways, ACCs are often incompletely resected. The complete resection rates in reported cases ranged from 42 to 57% [10]. Negative surgical margins are difficult to obtain because of the relative inability to resect more than 6 cm of the trachea, and thus are prone to recur locally [6]. However, previous studies have found that overall survival rates of completely and incompletely resected ACCs were not significantly different, even when a residual tumor was left behind after surgical resection [11, 12]. Maziak showed a five-year cumulative survival rates for patients with complete and incomplete resection were 82% versus 77% [3]. In contrast Gaissert et al [13] showed statistically significant longer DFS with negative airway margins. The lack of statistical significance on survival benefit for negative margins in other studies is likely due to low numbers and inadequate follow-up. ACC seems to possess radiosensitivity, most tumors respond to radiotherapy wich often results in long periods of remission for patients treated with radiotherapy alone [5].

When the tumor is considered unresectable due to its local extension, exclusive radiotherapy is given at dose of 66 to 70 Gy in conventional fractionation [7]. Because ACCs often traverse long regions throughout the thorax, especially in the vertical direction, margins of 4-5 cm in craniocaudal axis were suggested by Kaminski and al [11]. Conformal radiation therapy is currently recommended. Primary radiotherapy has had mixed results, with a locoregional control rate between 20% and 70% [14]. Maziak et al [3] reported that patients who initially received radiotherapy alone had a mean survival time of 37 months, ranging 4 months to 7 years. In fact our first patient was unresectable because of the extent of the tumor from the carina into proximal left main-stem bronchus, and survival at 7 years was noted after a radiotherapy dose to 66 Gy. In the largest series, Gaissert et al [13] reviewed 135 patients with tracheal ACC and showed a 52% and 29% 5- and 10-year survival, respectively, in resected ACC, but only a 33% and 10% 5- and 10-year survival in unresectable ACC of the trachea. Neutron radiotherapy has been shown to be effective in advanced unresectable ACC of the trachea in a recent study by Bittner et al [12], they showed a 5-year overall survival rate of 89% and a 5-year locoregional control rate of 54% in their cohort of 20 patients with unresectable tracheal ACC treated with upfront neutron radiotherapy.

The role of post-operative adjuvant radiotherapy remains uncertain. Some advocate a systematic radiotherapy while others only offer this treatment when margins are invaded [15, 16]. In adjuvant situation the overall survival rate at 5 and 10 years were respectively in three major series 66-79% et 51-57% [3, 16, 17]. We realized a post operative radiotherapy; in case 2; because the upper surgical margin was narrow; only at 3 mm. With limitations of randomized comparison, it stills seems reasonable to recommend adjuvant radiotherapy for all patients undergoing resection, and certainly for those in whom the final pathologic examination identifies residual tumors at the resection margins. A period of at least one month is recommended after surgery and it may be useful to perform a bronchosccopy to ensure healing. Post-operative radiotherapy is recommended at 45 to 65 Gy, depending on the quality of margins. According to the results of the few investigations, chemotherapy does not appear to have a role in the treatment of ACC of the trachea [1].

## Conclusion

ACC is a rare primary tracheal malignancy. This disease is commonly misdiagnosed as asthma. Surgical resection followed by radiotherapy is widely recommended protocol for treatment of localized tracheal tumors and provides the best chance of pronlonged survival. Patients with unresectable disease, can benefit long periods of remission due to the use of conformal radiotherapy; wich allows the delivery of high dose with an acceptable level of morbidity.
